# Paradigm shift in infection control practices in dental clinics in response to COVID-19 among dental professionals in Thailand

**DOI:** 10.3389/froh.2022.979600

**Published:** 2022-09-21

**Authors:** Phisut Amnuaiphanit, Thanasak Thumbuntu, Piyada Gaewkhiew, Ruchanee Salingcarnboriboon Ampornaramveth

**Affiliations:** ^1^ Dental Department, Kapho Hospital, Pattani, Thailand; ^2^ Dental Division, Royal Thai Army Medical Department, Bangkok, Thailand; ^3^ Department of Community Dentistry, Faculty of Dentistry, Mahidol University, Bangkok, Thailand; ^4^ Center of Excellence on Oral Microbiology and Immunology, Department of Microbiology, Faculty of Dentistry, Chulalongkorn University, Bangkok, Thailand

**Keywords:** infection control (IC), dental clinic, COVID-19, dental health care professionals, Thailand

## Abstract

Infection control (IC) practice routines depend mainly on knowledge, perception, and awareness of a disease among dental professionals. However, there has been no report on the perception, awareness, and adaptability to the new practice guidelines of Thai dental professionals (dentists, dental nurses, dental assistants, and dental technicians) to the COVID-19 pandemic. This study aims to investigate how dental professionals in Thailand perceive and are aware of COVID-19, and how they have changed their IC practices in response to the pandemic. Online cross-sectional surveys using convenience sampling during September 2021 were sent to Thai dental professionals. The data were analyzed using descriptive statistics and the Chi-square test. Statistical analysis was performed using the Statistical Package for Social Sciences, version 22.0. The tests were two-tailed, with a significance level of *p* < 0.05 and 95% confidence intervals (CIs). The 1,177 dental professionals who completed the questionnaire were from the public and private sectors. Most respondents obtained their knowledge about COVID-19 from social media (91.8%). 86.7% had adapted to the new IC practice guidelines. The respondents reported that they had modified their work practices in several aspects; changes in administrative control, 1,039 (88.3%); enhancing local source control of dental aerosols, 1,031 (87.6%); heightening sterilization and disinfection procedures, 1,032 (87.7%); and improving the ventilation system, 994 (84.5%). As of October 2021, 1,162 (98.7%) respondents were vaccinated, and 47 (3.99%) had tested positive for COVID-19 compared with 2.30% in the general population. Among infected individuals, 10 (21.3%) were suspected of being infected while working in the dental setting. In conclusion, with an average worry score well over 4.10 out of 5, more than 96% of Thai dental professionals reported seeking updated knowledge and agreed that escalation of IC measures was needed. However, only 86.7% improved their COVID-19 infection prevention practices in 4 aspects and appropriate PPE use. The infection rate in dental professionals was 3.99%, with the highest infection rate in dental assistants. Despite statistical insignificance of infection rate between changed and unchanged group, it cannot be concluded that stricter IC measures are negligible as ones might contract disease from setting other than work.

## Introduction

The emergence of the severe acute respiratory syndrome coronavirus 2 (SARS-CoV-2), which causes COVID-19, raises serious concerns about infection control practices, especially among dental professionals. COVID-19 can be transmitted from human to human by multiple means, droplets, aerosols, and fomites (1). It has been confirmed that SAR-CoV-2 can be transmitted *via* airborne particles from symptomatic, asymptomatic, and presymptomatic patients (2, 3). Coronavirus has a diameter between 50 and 200 nm that can be carried in aerosols 5 µm or smaller (4, 5). In the dental clinic, regular interpersonal activities and most dental treatments utilizing rotary dental instruments in the oral cavity, which is enriched with saliva and respiratory droplets, the so-called aerosol-generating procedures (AGPs), are a concern. An AGP can generate particles of different sizes, ranging from droplets to aerosols.

Prior to the COVID-19 pandemic, standard precautions were the main infection control strategies used to control disease transmission in the dental clinic. In Thailand, they were incorporated into the Dental Safety Goals and Guidelines 2015 and the dental clinic accreditation scheme by the Dental Council of Thailand (6). However, these protocols are not sufficient for preventing airborne diseases. Therefore, dental infection control practices needed an escalation to meet the challenge of COVID-19 transmission.

Several studies have investigated the perceptions, concerns, and adaptability of dental health care personnel to the COVID-19 pandemic (7–9). In response to the pandemic, the US Centers for Disease Control and Prevention (CDC) released the Interim Infection Prevention and Control Guidance for Dental Settings during the COVID-19 Response, which were adopted and adapted as new guidelines in many countries, including Thailand (10). The protocols for dental practices in Thailand during COVID-19 were launched by the Department of Medical Services, Ministry of Public Health in collaboration with the Dental Council of Thailand, the Royal College of Dental Surgeons of Thailand and other relevant organizations (11–13). Compared to a pre-existing guideline, the new one focused on management according to patients' necessity and severity—emergency, urgency, and elective dental procedures. It additionally covered a few perspectives of IC practices. Firstly, administrative control such as patient screening, shifting towards an appointment system, and physical distancing measures were encouraged. Secondly, environmental management was strongly recommended, such as local source control by pre-procedural rinsing together with using rubber dam and high-power suction. Enhancing ventilation system was also encouraged for all facilities. Lastly, clearly defined levels of PPE were delineated concerning patient's and operation's risk.

However, there are no binding regulations in Thailand that require a clinic to strictly follow. The willingness of the dental professionals to change their infection control practice routines depends mainly on their knowledge and awareness of the disease. Currently, there is no report on the perception, concern, and adaptability to the new practice guidelines due to the COVID-19 pandemic in Thai dental personnel. The primary aim of this study was to determine how dental professionals in Thailand perceive and are aware of COVID-19, and how they have changed their infection control practices in response to the pandemic. Secondly, the prevalence of COVID-19 infection among dental professionals was also identified in this cross-sectional survey.

## Materials and methods

### Study design

The study was conducted as an online cross-sectional survey using convenient sampling during September 2021. The dental professionals in public, educational, and private facilities were electronically invited to participate. A link to the questionnaire was sent to the participants, together with a brief written introduction to the background, the study's objective, voluntary nature of participation, declarations of confidentiality and anonymity, a right to withdraw from survey, and instructions for completing the questionnaire. Participating dental professionals read the text and pressed the confirm button to express their consents. Without consent from the participants, the online form automatically terminated. The Ethics Committee of the Faculty of Dentistry, Chulalongkorn University, Thailand has approved the study to be carried out according to the protocol and informed permission dated and/or amended as follows in compliance with the ICH/GCP (HREC-DCU2021-110).

### Sample size calculation

The online Raosoft sample size calculator was used to calculate the appropriate sample size for this study. Among dental professionals in Thailand, 18,965 dentists, 7,508 dental nurses, and an estimated 28,448 dental assistants are actively practicing. The required sample size was 382 per dental professional level with a confidence level of 95% and a 5% margin of error. The response acceptance was closed (October 3, 2021) when the required sample size was achieved.

### Questionnaire

The Thai language questionnaire was designed using Google forms. The researchers set every question as mandatorily required field which participants could not proceed and submit without answering. A link was shared with the participants *via* social media, i.e., Line application groups and Facebook. The participants typically completed the survey in ∼5 min. The survey questions were adopted the content following the guidelines from the Department of Medical Services, Ministry of Public Health for dental practice during the COVID-19 situation in 2021. It was piloted among 20 dental health practitioners. All feedbacks and unclear contents, including evaluating whether each of the questions matches the concept, were amended to cover all points, and retested on the same group. The questionnaire comprised four main topics; socio-demographic data; perception and awareness of the dental professionals about COVID-19 represented by worry score and information seeking behavior; modification of infection control routines to match the new recommendation and problems in following the new recommendation; and prevalence of contracting COVID-19, the potential cause of infection, and vaccination history in dental professions.

### Statistical analysis

The data are presented as frequency with percentages for categorical variables. The normal distribution of the data was assessed *via* box-plot and central tendency measurements.

The Mann-Whitney *U* test and the Kruskal–Wallis test were used to identify the difference of worry score in different sector and cadres of dental professionals. The association between changing in IC practices and perception—in term of worry score, and awareness—in term of knowledge seeking behavior were analyzed by logistic regression. Chi-square test was used to compare the compliance to guideline among different sectors and cadres. The association between changing in IC practices and different professional roles were identified by logistic regression. Statistical analysis was performed using the Statistical Package for Social Sciences, version 22.0. The tests were two-tailed, with a significance level of *p* < 0.05 and 95% confidence intervals (CIs).

## Results

**Figure 1:**
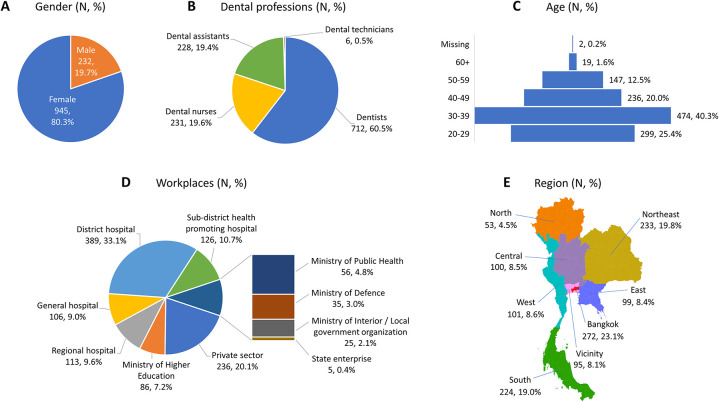
Respondent demographics classified by (**A**) sex, (**B**) dental personnel level, (**C**) age profile, (**D**) workplace, and (**E**) region (*N*, %).

This study was conducted in the middle of Thailand's fourth wave of the COVID-19 pandemic. The data were collected for approximately one month, from September 1 to October 3, 2021. When this study was conducted, there were 432,703 cases of COVID-19 in Thailand (13,112.21 cases/day on average), accounting for 0.62% of the Thai population (69.8 million). Demographic data of the 1,177 dental professionals completing the questionnaire is shown in Figure 1.

Dental professionals from the public and private sector expressed concern about the COVID-19 pandemic with a mean worry score of 4.07 and 4.23, respectively (*p* = 0.057) which was not statistically significant. Significantly different worry scores were observed in the different professional cadres; higher scores were observed in dental technicians, dental assistants, and dental nurses compared with dentists (Table 1).

**Table 1:** Worry score and types of occupation roles (*N* = 1177).

Profession role	Mean worry score ± S.D.	Coeff^a^	95%CI^a^	*p*-value^b^
Dentist	4.02 ± 0.84	Ref	Ref	*<0.001^#^*
Dental nurse	4.23 ± 0.88	0.53	[0.25,0.81]*	
Dental assistants	4.20 ± 0.92	0.47	[0.19, 0.76]*	
Dental technician	4.67 ± 0.52	1.48	[−0.17, 3.13]	

SD, standard deviation.

^a^ Ordered logistic regression.

^b^ Kruskal Wallist test.

* *p* < 0.05.

Among the 1,177 respondents, 1,125 (95.6%) paid attention to news about COVID-19 pandemic. 1,164 or 98.9% actively sought additional knowledge and up-to-date infection control protocol. Social media (1,081; 91.8%), guidelines launched by professional institute (906; 77.0%), academic meeting (418; 35.9%) were the major sources for new knowledge on infection control practice. The perception (worry score) was statistically significant associated with changing infection control protocol including change in administrative control, sterilization and disinfection and improved ventilation system in clinic. While only sterilization was associated with awareness (updating knowledge) (*p* < 0.05) (Table 2).

**Table 2:** Association between perception and infection control protocol was analyzed both in crude and adjusted models through logistic regression analysis (*N* = 1177).

	Perception (Worry score)						Awareness (Seeking updated knowledge about IC guidelines)					
Infection control practice	Crude			Adjusted^a^			Crude			Adjusted^a^		
	OR	95%CI	*p* for trend	OR	95%CI	*p* for trend	OR	95%CI	*p* for trend	OR	95%CI	*p* for trend
Changing IC protocol for working	0.72	[0.58, 0.89]	0.002*	0.75	[0.61, 0.93]	0.007*	0.79	[0.36, 1.73]	0.768	0.45	[0.06, 3.13]	0.417
Change in dministrative control	0.86	[0.76, 0.97]	0.012*	0.86	[0.76, 0.97]	0.013*	1.09	[0.71, 1.67]	0.699	1.26	[0.82, 1.93]	0.297
Enhancement of local source control of dental aerosols	0.91	[0.80, 1.02]	0.106	0.89	[0.79, 1.00]	0.054	1.29	[0.86, 1.95]	0.221	1.35	[0.89, 2.05]	0.153
Sterilization and disinfection	0.81	[0.72, 0.92]	0.001*	0.82	[0.72, 0.93]	0.002*	1.54	[1.02, 2.32]	0.041*	1.58	[1.05, 2.39]	0.029*
Improved ventilation system in clinic	0.84	[0.75, 0.94]	0.004*	0.84	[0.74, 0.94]	0.004*	0.93	[0.61, 1.41]	0.732	1.04	[0.68, 1.58]	0.861
Personal protective equipment uses												
N95 respirator	0.88	[0.75, 1.03]	0.107	0.89	[0.75, 1.04]	0.134	1.18	[0.69, 2.03]	0.547	1.40	[0.81, 2.43]	0.229
KN95 face mask	0.91	[0.79, 1.04]	0.160	0.90	[0.78, 1.03]	0.121	1.08	[0.66, 1.76]	0.760	1.02	[0.62, 1.66]	0.942
Surgical mask with mask fitter or micropore tape	1.10	[0.96, 1.26]	0.151	1.07	[0.93, 1.23]	0.351	1.21	[0.74, 2.00]	0.447	1.01	[0.61, 1.68]	0.976
Surgical mask only	0.68	[0.57, 0.82]	<0.001*	0.68	[0.56, 0.82]	<0.001*	0.54	[0.24, 1.17]	0.118	0.51	[0.23, 1.12]	0.093

OR, odd ratio; IC, infection control.

^a^ Adjusted with age, gender, and type of profession role.

Logistic regression, *p<0.05.

**Figure 2:**
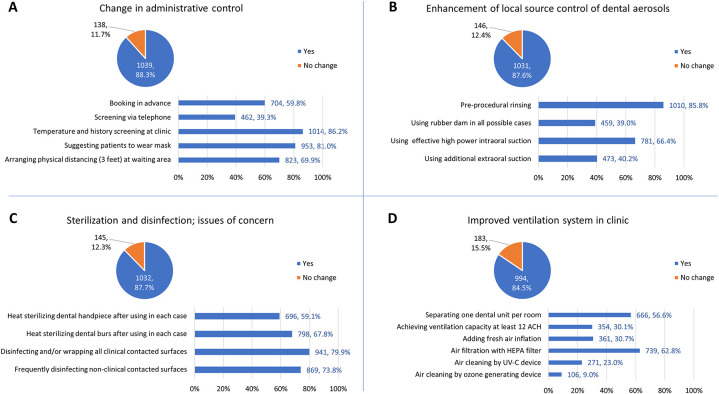
Changes in administrative control (**A**), local source control of dental aerosols (**B**), sterilization and disinfection practice (**C**), and ventilation system in the clinic (**D**) (*N*, %). A large and similar proportion of respondents, 85%–88%, reported change in these measures. Among those reported no change in all four mentioned measures, 136 participants suspended their operations instead of continuing under modified systems.

1,137 (96.6%) agreed with the need to change their clinical practices and environmental settings in response to the COVID-19 pandemic. 1,021 (86.7%) reported changing their practices to follow the new guidelines after the first wave of the COVID-19 pandemic in Thailand. The compliance of dental professionals to infection control practices, summarized in Figure 2, are divided into four main topics which are (1) change in administrative control (2) enhanced local source control of dental aerosols (3) sterilization and disinfection; issues of concern, and (4) improved the ventilation system in the clinic. For administrative control, temperature and history screening were implemented widely at 86.2%, while telephone screening was a less popular measure, implemented relatively low by 39.3% of respondents. Regarding local source control of dental aerosols, pre-procedural rinsing was implemented mainly (85.8%), followed by high power suction (66.4%), while extra-oral suction and rubber dam were less adopted at around 40%. For sterilization and disinfection, disinfecting or wrapping clinical contacted surfaces was achieved by 79.9%, while non-clinical contacted surfaces were frequently disinfected by 73.8%. A smaller proportion routinely performed heat sterilization of dental burs and handpieces, at 67.8% and 59.1%, respectively. For ventilation measure, the widely accepted measure was air recirculation through HEPA filter, implemented by 62.8%. In contrast, achieving air ventilation at 12 ACH was compiled by less than one-third.

**Figure 3:**
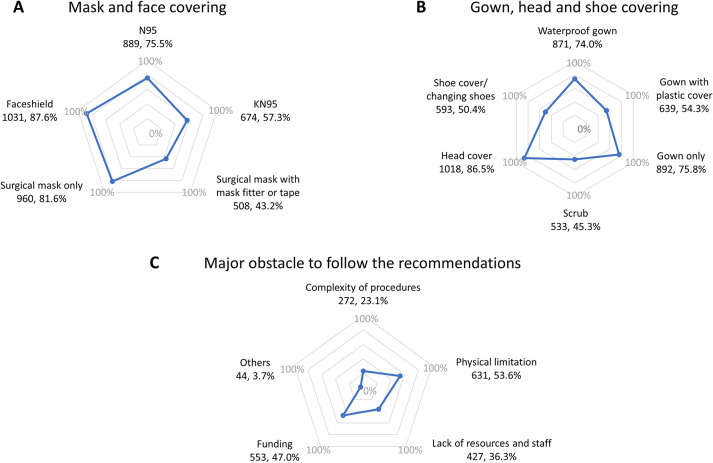
Utilization of personal protective equipment: mask and face covering (**A**); gown, head, and shoe covering (**B**). Major obstacle to following the recommendations (**C**) (*N*, %).

We found that 889 (75.5%) and 1,031 (87.6%) out of 1,177 respondents wore an N95 respirator and face shield, respectively. Due to the shortage of N95 respirators, some respondents, 674 (57.3%), chose to wear the cheaper and more accessible KN95 instead, and some respondents, 508 (43.2%), sealed the surgical mask with a custom-made mask fitter or micropore tape (Figure 3A). Wearing a waterproof gown 871 (74.0%), plastic suit over a gown 639 (54.3%), or wearing surgical scrubs 533 (45.3%) became trendy in dental clinics in Thailand (Figure 3B). The major obstacles in guideline compliance were the physical limitation of setting 631 (53.6%) (Figure 3C). The adaptability of the dental professionals to the new infection control guidelines due to the COVID-19 pandemic is summarized in Table 3. Additionally, the findings also showed the changing of protocol for working was significantly associated with different occupational role. Specifically, administrative control change, air ventilation change, N95 use and surgical mask with mask fitter use showed significant associated with different type of occupational role (*p* < 0.05). Dental assistant reported statistically significant lower chance to change protocol for working, and administrative control compared to dentist (OR < 1, *p* < 0.05). While enhancement of local source control of dental aerosols was higher than dentists (OR : 1.65, *p* < 0.05), but there was no significant difference between occupation roles (*p* > 0.05) (Table 4).

**Table 3:** Practice following infection control guidelines (*N* = 1177).

** **	Public *N* (%)	Private *N* (%)	*p*-value^a^
Dental service during April 2021 wave			<0.001*
** **Normal service	99 (10.5)	87 (36.9)	* *
** **Only aerosol controllable procedure (such as simple extraction, scaling using hand instruments, and treating caries lesions with Atraumatic Restorative Treatment)	350 (37.2)	98 (41.5)	* *
** **Only emergency/urgency	380 (40.4)	27 (11.4)	* *
** **Suspend	112 (11.9)	24 (10.2)	* *
Change in administrative control			
** **Booking in advance	536 (57.0)	168 (71.2)	*<0*.001*
** **Screening *via* telephone	328 (34.9)	134 (56.8)	*<0*.001*
** **Temperature and history screening at clinic	809 (86.0)	205 (86.9)	*0*.*723*
** **Suggesting patients to wear mask	749 (79.6)	204 (86.4)	*0*.017*
** **Arranging physical distancing at waiting area	641 (68.1)	182 (77.1)	*0*.007*
Enhancement of local source control of dental aerosols			
** **Pre-procedural rinsing	799 (84.9)	211 (89.4)	*0*.*077*
** **Using rubber dam in all possible cases	390 (41.5)	69 (29.2)	*0*.001*
** **Using effective high power intraoral suction	616 (65.5)	165 (69.9)	*0*.*195*
** **Using additional extraoral suction	353 (37.5)	120 (50.9)	*<0*.001*
Sterilization and disinfection			
** **Heat sterilizing dental handpiece after using in each case	577 (61.3)	119 (50.4)	*0*.002*
** **Heat sterilizing dental burs after using in each case	645 (68.5)	153 (64.8)	*0*.*275*
** **Disinfecting and/or wrapping clinical contacted surfaces	748 (79.5)	193 (81.8)	*0*.*432*
** **Frequently disinfecting non-clinical contacted surfaces	684 (72.5)	185 (78.4)	*0*.*075*
Improved ventilation system in clinic			
** **Separating one dental unit per room	506 (53.8)	160 (67.8)	*<0*.001*
** **Achieving ventilation capacity at least 12 ACH	298 (31.7)	56 (23.7)	*0*.017*
** **Adding fresh air inflation	306 (32.5)	55 (23.3)	*0*.006*
** **Air filtration with HEPA filter	572 (60.8)	167 (70.8)	*0*.005*
** **Air cleaning by UV-C device	164 (17.4)	107 (45.3)	*<0*.001*
** **Air cleaning by ozone generating device	69 (7.3)	37 (15.7)	*<0*.001*
Personal protective equipment uses			
** **N95 respirator	704 (74.8)	185 (78.4)	*0*.*253*
** **KN95 face mask	558 (59.3)	116 (49.2)	*0*.005*
** **Surgical mask with mask fitter or micropore tape	428 (45.5)	80 (33.9)	*0*.001*
** **Surgical mask only	780 (82.9)	180 (76.3)	*0*.019*
** **Faceshield	820 (87.1)	211 (89.4)	*0*.*345*
** **Waterproof gown	702 (74.6)	169 (71.6)	*0*.*349*
** **Gown with plastic cover	511 (54.3)	128 (54.2)	*0*.*985*
** **Gown only	731 (77.7)	161 (68.2)	*0*.002*
** **Scrub	433 (46.0)	100 (42.4)	*0*.*315*
** **Head cover	812 (86.3)	206 (87.3)	*0*.*689*
** **Shoe cover/changing shoes	487 (51.8)	106 (44.9)	*0*.*060*

ACH, air changes per hour.

^a^ Chi-square.

*statistically significant.

**Table 4:** Association between occupation role and infection control practice changes was analyzed both in crude and adjusted models through logistic regression analysis (*N* = 1177).

Infection control practice	Crude			Adjusted^a^		
	OR	95%CI	*p*-value	OR	95%CI	*p*-value
Changing IC protocol for working			*0.098*			*0.020^#^*
Dentist	Ref	Ref	* *	Ref	Ref	* *
Dental nurse	0.92	[0.39, 2.22]	* *	0.60	[0.22, 1.65]	* *
Dental assistant	0.48	[0.23, 0.98]*	* *	0.39	[0.19, 0.83]*	* *
Dental technician	1	–	* *	1	–	* *
Change in administrative control			*<0.001^#^*			*<0.001^#^*
Dentist	Ref	Ref	* *	Ref	Ref	* *
Dental nurse	0.28	[0.21, 0.36]*	* *	0.33	[0.24, 0.45]*	* *
Dental assistant	0.70	[0.53, 0.91]*	* *	0.72	[0.55, 0.95]*	* *
Dental technician	0.68	[0.14, 3.32]	* *	0.62	[0.12, 3.14]	* *
Enhancement of local source control of dental aerosols			*0.449*			*0.928*
Dentist	Ref	Ref	* *	Ref	Ref	* *
Dental nurse	0.27	[0.21, 0.36]*	* *	0.27	[0.20, 0.38]*	* *
Dental assistant	1.65	[1.26, 2.18]*	* *	1.64	[1.24, 2.18]*	* *
Dental technician	0.55	[0.10,2.89]	* *	0.43	[0.08, 2.30]	* *
Sterilization and disinfection			*0.032^#^*			*0.056*
Dentist	Ref	Ref	* *	Ref	Ref	* *
Dental nurse	0.33	[0.25, 0.44]*	* *	0.29	[0.21, 0.39]*	* *
Dental assistant	1.25	[0.93, 1.66]	* *	1.19	[0.88, 1.59]	* *
Dental technician	0.46	[0.11, 1.96]	* *	0.37	[0.09, 1.53]	* *
Improved ventilation system in clinic			*<0.001^#^*			*0.036^#^*
Dentist	Ref	Ref	* *	Ref	Ref	* *
Dental nurse	0.22	[0.17, 0.30]*	* *	0.24	[0.18, 0.33]*	* *
Dental assistant	1.12	[0.86, 1.46]	* *	1.16	[0.88, 1.51]	* *
Dental technician	0.56	[0.13, 2.31]	* *	0.53	[0.13, 2.20]	* *
Personal protective equipment uses						
N95 respirator				*<0*.*001^#^*		*<0*.*001^#^*
Dentist	Ref	Ref		Ref	Ref	
Dental hygienist	0.19	[0.14, 0.27]*		0.42	[0.25, 0.69]*	
Dental assistant	1.08	[0.72, 1.60]		0.84	[0.55, 1.29]	
Dental technician	0.43	[0.08, 2.37]		0.38	[0.06, 2.27]	
KN95 face mask			*0*.*052*	* *		*0*.*147*
Dentist	Ref	Ref		Ref	Ref	
Dental hygienist	0.51	[0.37, 0.68]*		0.83	[0.54, 1.30]	
Dental assistant	2.20	[1.57, 3.06]*		1.85	[1.30, 2.63]*	
Dental technician	1.50	[0.27, 8.24]		1.29	[0.22, 7.63]	
Surgical mask with mask fitter or micropore tape			*<0*.*001^#^*	* *		*<0*.*001^#^*
Dentist	Ref	Ref		Ref	Ref	
Dental hygienist	1.38	[1.02, 1.87]*		1.83	[1.18, 2.83]*	
Dental assistant	3.00	[2.20, 4.09]*		2.99	[2.14, 4.19]*	
Dental technician	1.75	[0.35, 8.73]		2.10	[0.39, 11.31]	
Surgical mask only			*0*.*692*	* *		*0*.*759*
Dentist	Ref	Ref		Ref	Ref	
Dental hygienist	0.44	[0.32, 0.62]*		0.75	[0.40, 1.39]	
Dental assistant	2.30	[1.38, 3.82]*		1.77	[1.04, 3.03]*	
Dental technician	1.04	[0.12, 9.02]		0.98	[0.10, 9.30]	

Ref, reference; OR, odd ratio; IC, infection control.

^a^ Adjusted with age, gender, and type of workplace.

Logistic regression, *p < 0.05.

^#^statistically significant.

**Figure 4:**
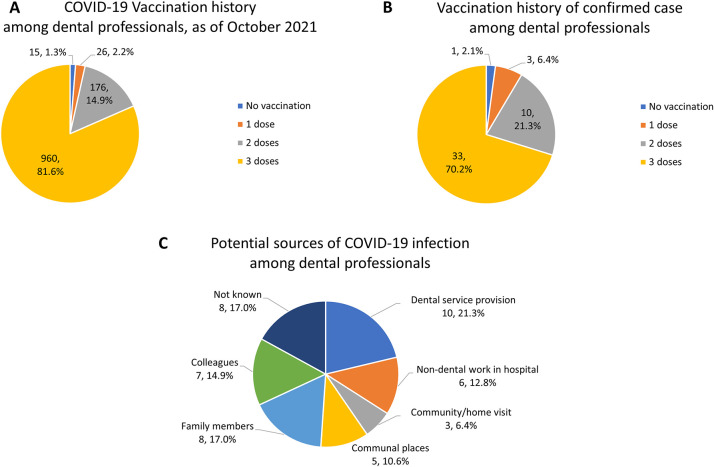
Vaccine taken up by the respondents (**A**) and by the COVID-19 confirmed case (**B**). Potential sources of COVID-19 infection among dental personnel (**C**) (*N*, %). As of October 2021.

As of October 2021, 1,162 (98.7%) out of 1,177 respondents in this study were vaccinated. Most of them, 960 (81.6%), were vaccinated with three vaccine shots, in which the first two doses were inactivated virus vaccines, the most available type in the country, and the third booster doses were mRNA vaccines. 176 respondents (14.9%) received two doses of vaccine, predominantly a viral vector or mRNA vaccine. The remaining 15 individuals (1.3%) had not received any vaccination (Figure 4A).

Out of the 1,177 dental professions enrolled in this study, 47 (3.99%) reported a positive test for COVID-19. Of these, 10 (21.3%) suspected that they became infected from patients while providing treatment, 6 (12.8%) from their non-dental work in hospitals, 8 (17.0%) from their family members, 7 (14.9%) from colleagues, 5 (10.6%) from places in everyday life, e.g., local market or accommodation, and 3 (6.4%) from their work related to the community or home visits. In comparison, 8 (17.0%) did not know the source of infection (Figure 4C). Among the 47 infected dental professions, only one person was unvaccinated (Figure 4B).

Comparing infection rate in three groups of standard compliance, dental professionals, who has not changed their protocols at all, reported highest rate of infection, standing at 5.4%. Lower infection rate was found in those with at least one change, accounting for 4.2% while the dental personnel who discontinued dental services reported the lowest infection rate at 3.8%. However, there was not shown the significant result in statistically in trend (*p* > 0.05).

## Discussion

To the best of our knowledge, this is the first report addressing the perception, awareness about COVID-19, compliance with infection control measures, and the prevalence of COVID-19 infection among Thai dental professionals. This survey was conducted when the COVID-19 situation in Thailand was in the middle of the fourth wave, which had a higher infection rate compared with the previous waves in 2020 (14). The strain identification, conducted through SNP genotyping by RT-PCR between 25 September to 1 October 2021 by the Department of Medical Sciences, Ministry of Public Health, discovered that more than 99% of infections were caused by the Delta variant (B.1.617.2) (15).

Thai dental professionals in the public and private sectors worried about the COVID-19 pandemic at a similar magnitude which conformed with several studies of fear and worry in the dental profession in many countries. A Norwegian study revealed that public and private practitioners’ fear of being infected and transmitting the disease to others was not significantly different. However, private practitioners were more concerned about making work-environment alterations than public dental professionals (16). These findings in the UK contrasted with those in Norway in that private practitioners were more worried compared with their public sector counterparts (17). Several studies reported that dental professionals were worried about spreading COVID-19 to their family members (16, 18).

Interestingly, in this study, the worry score differed among the dental personnel levels. Dental technicians, dental nurses, and dental assistants were more anxious than dentists. Unlike in Thailand, the Norwegian study found no significant difference in the worry scores between dental professional levels providing services during the lockdown (16). Notably, the Thai survey revealed that anxiety about the situation was lower in a more sophisticated dental personnel level. Knowledge about the disease and how to effectively prevent it can reduce the adverse psychological stress for dentists. Moreover, financial and resource insecurity and job loss in case of infection might be sources of concern, requiring support in the workplace and systematic preparedness for subsequent emerging infectious diseases.

It is noteworthy that perception and awareness of COVID-19 alone might not lead to change in IC practices. Although over 96% of responders sought knowledge and acknowledged the necessity to strengthen IC measures, only 86.7% realized change. Physical limitation of setting (53.6%) and funding restraints (43.7%) were reported as potential barriers.

Public dental services delivered during COVID-19 were limited to emergency or urgent treatment. In contrast, private dental practices accounted for a higher proportion of delivering normal services mostly limited to aerosol controllable procedures. This compliance discrepancy was observed in Thailand and seven other countries, as demonstrated in a 36-country survey conducted by the COVIDental Collaboration Group (2021) (9).

Temperature and history screening were frequently performed at both public and private facilities, which is congruent with an international trend that 57.6% of dental personnel enacted this measure (9). Pre-procedural antimicrobial rinsing and using high power intraoral suction were used equally in both private and public facilities. Rubber dams were used more commonly in public practices compared with private practices while the latter group used extraoral suction more often.

Several studies have confirmed the effectiveness of mouth rinses, such as povidone-iodine, chlorhexidine, hydrogen peroxide, could reduce the SARS-CoV-2 viral load (19). Seneviratne et al. also revealed that 30 s of gargling with 5 ml 0.5% povidone-iodine mouthwash showed effectively reduced SARS-CoV-2 levels in saliva for six hours (20). More than 50% of dental professionals used chlorhexidine, and 5%–28% used povidone-iodine (21).

A rubber dam together with pre-procedural rinsing substantially reduces bacterial contamination by 99.4%, compared with the conventional procedure without rubber dam application (22, 23). A multinational study found that over 85% of dental professionals recognized the importance of rubber dam application in reducing the chance of contaminated aerosols (24). In contrast, only 14% of dentists applied a rubber dam on every patient (25). Only 39.0% of Thai dental professionals reported using rubber dams in their practices. The barriers to compliance were the unavailability of rubber dams at the workplace, difficulty in using them, and being time-consuming (26), while the cost might not be a barrier (27).

Deploying high-volume evacuators together with pre-procedural rinsing substantially reduced bioaerosols, compared with one measure alone (28). A multinational study reported that 76% of dental professionals used high power evacuator (25). Using a high-volume evacuator with an aerosol cannula at a suction capacity of 150 mmHg and an airflow rate of 325 liters per minute can considerably reduce SARS-CoV-2-contaminated aerosols from ultrasonic scalers and high-speed handpieces (29).

The effectiveness of using extraoral suction is unresolved. Some studies reaffirmed the efficacy of extraoral suction in reducing contaminants (30, 31). However, more recent studies noted that its effectiveness was limited to the area close to the device and depended on suction positioning and the distance from the source of the contaminants (32, 33).

Although sterilizing dental handpieces in an autoclave is recommended in the current infection control guidelines (34), some dental clinics cannot afford to follow the guidelines and maintain using chemical disinfection. Our study demonstrated that public dental offices reported significantly higher compliance in handpiece sterilization compared with private offices.

Adapting to the Guideline for Environmental Infection Control in Healthcare Facilities (35), the Thai dental authority established a minimum of 12 ACH as the recommended standard for the environment in the dental clinic. This requirement was met more in the public than the private sector. This is partly because the public sector provided funds for ventilation improvement to most public dental facilities.

Using PPE heightened the protection of dental staff's safety. We found no significant difference in wearing an N95 respirator between private and public dental personnel. The compliance of Thai dental personnel in wearing an N95 and face shield can be considered relatively high compared with the international behavior surveyed by the COVIDental Collaboration Group. The study reported that, in most countries, over 50% of the dental workforce used an N95, and 41.07% wore eye protective equipment (9).

Self-reported infection rate in dental professionals, standing at 3.99%, was higher than infection rate of 2.30% of general population (14, 36). Broken down by professional type, infection rate of dental assistants and dental nurses were higher than the general population, standing at 13.16% and 3.99%, respectively. Conversely, dentists reported lower rate of infection, standing at only 1.12%, which conformed to most countries in the study of the COVIDental Collaboration Group (9). One death case was reported in the news during May 2021 (37). However, it should be noted that there is no official report from the organization to specify only the death of dental professionals related to COVID-19 except for this news. The exact number might be higher.

In Thailand, the lower prevalence in dentists can be explained by four major factors. The administrative measures by limiting service provision to only urgent care or aerosol controllable procedures, was implemented by over 80% of the respondents. Furthermore, 86.7% of Thai dental professionals enacted multiple infection control measures simultaneously to increase the success rate in infection prevention and control based on “A Swiss Cheese Model” and national guidelines. In addition, most dentists used an N95 or equivalent which associated with the lower infection rate of dentists in many countries (9). Lastly, 81.6% vaccination coverage with three doses might be vital in infection prevention.

However, the COVID-19 prevalence in dental assistants was much higher than the general population even if they operated in similar workplaces and had same vaccination as dentists. These findings among dental assistants were contrary to France study which the infection rate among dental assistants was lower than dentists and general population (38). The scenario in the Thai case can be explained by two reasons: respirator donning and doffing and allocation policy. Because there is no formal training in N95 donning and doffing for dental assistants, its appropriate use might be inadequate. Furthermore, the allocation of N95 based on limiting costs might influence dental assistants to use the PPE for up to 1 week or until soiled or damaged. These factors might have reduced the effectiveness of protection and increased the risk of contamination.

Despite statistical insignificance of infection rate between changed and unchanged group, dental professional with reported changes in every aspect had the lowest infection rate, compared to groups with partial change and no change, respectively. It is likely that conforming to new standards might reduce the chance of contracting COVID-19 to a certain extent. However, COVID-19 infection could depend on several factors including social bubbles, daily lifestyle, and immunization, therefore, the statistical insignificance could not be used as a justification to future incompliance to the standard.

Among countries in South and Southeast Asia, inconsistent dental services during the COVID-19 pandemic were reported. In the study of COVIDental Collaboration Group, Singaporean dental services, both private and public facilities, provided routine dental services. On the other hand, public and private Indian facilities only delivered urgent dental services. Malaysia reported a combination model of response. The public dental services only provided services to urgent cases while private dental practices continued their routine services. In addition to the changing IC protocol, it is noteworthy that having additional measures, such as phone screening prior to dental visit, surface disinfection and proper PPE use, might reduce the chance of infection in dental professionals. In India and Singapore where majority of dental professionals implemented these measures, infection rates of dental personnel were lower than the general population, while in Malaysia where mentioned measures were adopted by lower proportion of dental professionals, the infection rate of professional group was four time higher than the general population (9).

During September 2021 in Vietnam, there was no specific guideline for dental services. Dental professional mainly relied on general IC protocol for medical facilities. Vaccination coverage in dental professionals was only 75.9% (39). In Indonesia, despite the recent surge of publications about COVID-19 and dental services, the regulators did not provide a clear guideline at the early stage. Among 24 Indonesian health professionals died from COVID-19, 25% were dentists (40, 41). Both Vietnam and Indonesia reported a shortage of PPE as a major hindrance in realizing personnel safety during COVID-19 pandemic (39, 42). In Cambodia and the Philippines, Ministry of Health and professional association played an important role in guideline dissemination and ensure the compliance of dental facilities, respectively (43–45). The structure and elements of guidelines of the Philippines covered screening, physical distancing, PPE use, local source control, air disinfection and other stricter IC measures which congruent with the Thai's guidelines (46).

The present study has several limitations. First, the questionnaire was tested and validated in a small group of intended respondents employing face validity. A more systematic way, such as a pilot survey and checking for internal consistency, should be used for questionnaire validation. The exact number of dental assistants in private facilities is not known because dental assistants are not required to register with state regulators in Thailand. Therefore, the population size of the dental assistants was estimated, using a proposed ratio of dentists to dental assistants as 1:1.5 (47). In addition, the number of dental technicians responding to this survey was too small for representation and inference. Moreover, the reliability about the potential sources of infection might be compromised because the respondents subjectively reported the attributable sources, and no further investigation was performed by the researchers. There is large body of research describing how to prevent COVID-19 transmission in the dental clinic. Future studies should focus on evaluating these infection control measures in terms of their effectiveness and efficiency to inform evidence-based prioritization of effective measure.

In conclusion, with an average worry score well over 4.10 out of 5, more than 96% of Thai dental professionals reported seeking updated knowledge about infection prevention and agreed that escalation of IC measures was needed. However, only 86.7% improved their COVID-19 infection prevention practices. The infection rate in dental professionals was 3.99%, with the highest infection rate in dental assistants, followed by dental nurses, and dentists, respectively. Despite statistical insignificance of infection rate between conformers and non-conformers, this could not be used as a justification for future incompliance to the standard as ones might contract disease from setting other than work.

## Data Availability

The raw data supporting the conclusions of this article will be made available by the authors, without undue reservation.
